# Large-Scale Serological Survey of Crimean-Congo Hemorrhagic Fever Virus and Rift Valley Fever Virus in Small Ruminants in Senegal

**DOI:** 10.3390/pathogens13080689

**Published:** 2024-08-15

**Authors:** Marie Cicille Ba Gahn, Gorgui Diouf, Ndjibouyé Cissé, Mamadou Ciss, Marion Bordier, Mbengué Ndiaye, Mame Thierno Bakhoum, Mamadou Lamine Djiba, Corrie Brown, Bonto Faburay, Assane Gueye Fall, Modou Moustapha Lo

**Affiliations:** 1 Institut Sénégalais de Recherches Agricoles, Laboratoire National de l’Elevage et de Recherches Vétérinaires (ISRA-LNERV), Dakar-Hann BP 2057, Senegal; 2 Centre de Coopération Internationale en Recherche Agronomique pour le Developpement (CIRAD), UMR ASTRE, F-34398 Montpellier, France; 3 LifeStock International, 550 Fortson Rd., Athens, GA 30606, USA; 4 Foreign Animal Disease Diagnostic Laboratory, National Veterinary Services Laboratories, National Bio and Agro-Defense Facility, United States Department of Agriculture, Manhattan, KS 66505, USA

**Keywords:** Crimean-Congo hemorrhagic fever, Rift Valley fever, serological survey, small-ruminant, Senegal

## Abstract

Crimean-Congo hemorrhagic fever (CCHF) and Rift Valley fever (RVF) are among the list of emerging zoonotic diseases that require special attention and priority. RVF is one of the six priority diseases selected by the Senegalese government. Repeated epidemic episodes and sporadic cases of CCHF and RVF in Senegal motivated this study, involving a national cross-sectional serological survey to assess the distribution of the two diseases in this country throughout the small ruminant population. A total of 2127 sera from small ruminants (goat and sheep) were collected in all regions of Senegal. The overall seroprevalence of CCHF and RVF was 14.1% (IC 95%: 12.5–15.5) and 4.4% (95% CI: 3.5–5.3), respectively. The regions of Saint-Louis (38.4%; 95% CI: 30.4–46.2), Kolda (28.3%; 95% CI: 20.9–35.7), Tambacounda (22.2%; 95% CI: 15.8–28.6) and Kédougou (20.9%; 95% CI: 14.4–27.4) were the most affected areas. The risk factors identified during this study show that the age, species and sex of the animals are key factors in determining exposure to these two viruses. This study confirms the active circulation of CCHF in Senegal and provides important and consistent data that can be used to improve the surveillance strategy of a two-in-one health approach to zoonoses.

## 1. Introduction

The emergence and/or re-emergence of vector-borne diseases (VBDs), strongly impacted by climate change, are a major public health problem [1]. Climate change is mainly determined by temperature, precipitation and environmental conditions, which can modify the risk of transmission of vector-borne diseases [2]. These factors play a decisive role in regulating vector development, survival and the reproductive behavior of vectors and larval habitats. Thus, global climate change may promote the development and colonization of vectors and the successful adaptation of vectors or pathogens to new ecosystems or previously unaffected areas [3,4]. Human and animal cases of mosquito and tick-borne diseases such as Rift Valley (RVF) and Crimean-Congo hemorrhagic fever (CCHF), respectively, have increased during recent years [5].

The CCHF virus (*Orthonairovirus* genus of the Bunyaviridae family) is an emerging virus circulating in over 30 countries across Africa, Europe and the Middle East [6]. The virus circulates in a silent, enzootic tick–vertebrate–tick cycle, and so far, there is no evidence that the virus causes disease in animals. The distribution of the CCHF virus correlates with the distribution of various species of hard ticks, such as those belonging to the Ixodidae family, particularly the *Hyalomma* genus, which is a biological vector and natural reservoir [7]. With global warming and climate change, the vector’s range is changing, altering the circulation of the virus and increasing the risk of its emergence in new geographical ecosystems [1]. Being a zoonotic disease, the main risk groups are farmers, veterinarians and slaughterhouse workers in endemic areas, and the most affected occupational groups are farmers and/or domestic workers and slaughterhouse workers. The second most affected group is medical staff in contact with infected patients using inappropriate personal protective equipment and processing inappropriate clinical practices [8,9]. From November 2015 to November 2019, human health services in Senegal confirmed eight human cases of CCHF, including four cases in 2019 in different geographical areas and three other cases imported from Mauritania, a neighboring country. Between April and November 2023, ten human cases of CCHF were also reported, with four human deaths [10]. Serological and virological evidence of CCHF in animals from Senegal [11,12,13] and the neighboring countries of Mauritania, Gambia and Mali [14,15,16,17] have been reported. In addition, a previous study showed that the seroprevalence of the CCHFV in humans was 0.7% in Senegal, and the specific IgG of the CCHFV was found in Kaolack, Diourbel, Fatick, Kaffrine and Saint-Louis regions [18]. The CCHFV was detected in ticks collected from livestock [19]. Bunyavirales are one of the largest viral orders, with over 350 viruses, including several viruses of public health and agricultural importance, such as the Crimean–Congo hemorrhagic fever virus (CCHFV) and the Rift Valley fever virus (RVFV) [20,21].

Rift Valley fever (RVF) is a mosquito-borne disease affecting mostly domestic animals in sub-Saharan Africa, such as cattle, buffalo, sheep, goats, camels and humans. The RVF virus belongs to the *Phlebovirus* genus of the Phenuiviridae family (order Bunyavirales). The disease is reported in most African countries and in the Middle East (Saudi Arabia and Yemen) [22,23]. Serological surveys show that the RVF virus was circulating in West Africa between 1981 and 1985, especially in southern Mauritania, with a prevalence of 18% among ruminants and 13% among ruminant breeders [24]. In 1987, the year in which a dam was built in the Senegal River at Diama (16°12′57″ N; 16°24′54″ O), outbreaks of RVF occurred for the first time in Mauritania and Senegal [25]. The RVF virus has been isolated four times from *Aedes d’alzieli* mosquitoes and once from a person with mild rotavirus syndrome in southeastern Senegal [26]. In the fall of 1987, a large RVF epizootic was reported in ruminants in the Senegal River valley, followed by human epidemics. Subsequent RVF epizootics associated, with human cases occurred in 1993, 1998 and 2003 in Senegal [27]. In recent years, the virus has been particularly active in Mauritania with outbreaks confirmed in 2010, 2012, 2015 and 2020, [28] while in Senegal, outbreaks were confirmed in 2012-2013 and 2014. A recent study showed that the seroprevalence of RVF was 3.94% in humans in Senegal [18]. Favorable environmental conditions, such as precipitation, are one of the main factors that cause unusual viral emergence in increased mosquito vector populations, leading to a large number of infected domestic animals, considered amplifying hosts.

Regarding these recurrent epidemics and sporadic CCHF and RVF cases in Senegal, it has become a real need to identify the favorable ecosystems of emergence and re-emergence of these two viruses to improve the response. Most studies of these two viruses have focused on the northern area of the country. The only nationwide implementation of CCHF and RVF seroprevalence studies in Senegal was conducted from the human side [18].

This study describes a cross-sectional serological survey that assesses the risk and maps the distribution of these two diseases in domestic small ruminant populations in Senegal.

## 2. Materials and Methods

### 2.1. Study Area and Samples Collection

**Figure 1:**
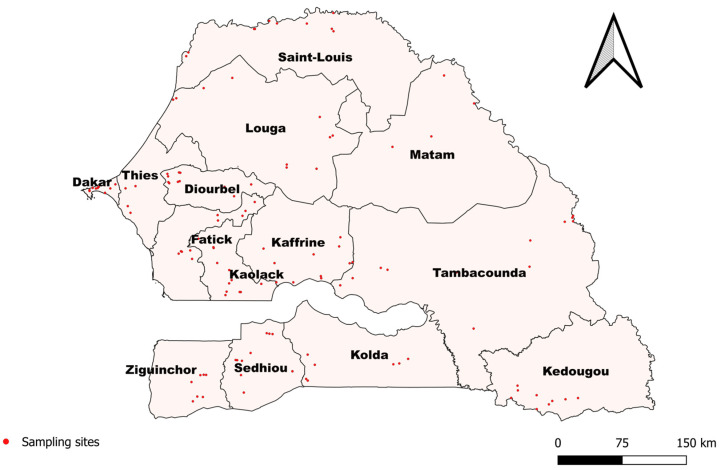
Map of sampling sites in 14 regions of Senegal.

A cross-sectional study was carried out between May and December 2023 to assess the presence of anti-RVFV immunoglobulins G (IgGs) and M (IgMs) and anti-CCHFV antibodies in small ruminants living in the fourteen regions of Senegal (Figure 1). The regions of Senegal are located between latitudes 12°8 and 16°41 north and longitudes 11°21 and 17°32 west. Consisting of three administrative departments each, the regions were grouped into several largely different agro-ecological areas. Each area had its own environmental characteristics. Livestock and farming practices vary according to each region, but the exposure of breeders and healthcare workers to RVF and CCHF seems similar. According to a previous investigation driven in various districts by the research institutes in Senegal, an expected animal prevalence of 10% was defined for both diseases, which led us to estimate a sample size of approximately 150 per region, with a precision of 10% using the Cochran formula. The number of samples was then distributed over two departments in each region and between various sampling sites in each department in proportion to their livestock density, according to the livestock census surveys carried out in 2020 by Senegalese veterinary services. To ensure good seroprevalence accuracy in each department, the number of sampling sites to be visited was calculated based on available RVF prevalence data. Animals were selected randomly from resident sheep and goat populations aged over three months. Preferential or guided sampling of animals was not applied in the field. To increase the probability of antibody detection, sites were selected because of their history of suspected or confirmed cases of CCHF and RVF and according to their ecosystems’ characteristics favorable for vector development.

Blood samples were collected by jugular vein puncture into dry gel tubes. The sera were collected after decantation and centrifugation at 8000× *g* for 5 min and were stored in a freezer at −20 °C and in coolers containing ice packs to maintain the cold chain up to the laboratory. For each animal, the following data were recorded: age (young/adult determined by animal’s teeth), species (goat/sheep), sex (male/female), and geographical location of the herd (geographic coordinates) provided by Garmin France OREGON 650 portable devices (10 m accuracy).

### 2.2. Serological Tests

Enzyme-Linked Immunosorbent Assay (ELISA) commercial ID.vet kits (Innovative Diagnostics, 34790 Grabels, France) were used to detect RVFV and CCHFV antibodies, respectively, in sera. The tests were carried out in accordance with the indications for the *ID Screen^®^ CCHF Double Antigen Multi-species* [29] and *ID Screen^®^ Rift Valley Fever Competition Multi-species* and *IgM capture* diagnostic kits designed to detect antibodies from the nucleoproteins (NPs) of CCHF and RVF viruses in the serum or plasma of small ruminants. Detection of anti-NP antibodies by ELISA indicates exposure to the virus through natural infection or vaccination. The samples were analyzed for RVF in two steps: an initial screening of all sera with the *Rift Valley Fever Competition Multi-species* kit was carried out to identify animals that had developed IgG (Immunoglobulin G) antibodies to the virus, indicating previous exposure to the virus, followed by a second step screening for *IgM* antibodies, indicating recent exposure to the virus. Results were reported in optical density (OD) with a threshold defining the positive or negative status of each animal according to the interpretation criteria recommended in each kit by the supplier.

### 2.3. Statistical Analyses

Statistical analysis was performed using R software [30] with R studio interface (version 4.3.1). True seroprevalence of infection was calculated by determining the percentage of positive samples within each variable category and according to the specificity (99.8% for RVF; 100% for CCHF) and sensitivity (100% for RVF; 98.9% for CCHF) of each kit. The association between RVFV and CCHFV prevalence and other variables was assessed through Chi-2 and logistic regression analysis. Statistical parameters through odds ratio (OR) and its 95% confidence intervals (CIs) were used to evaluate the relative risks between each variable (age, sex, species, and region). The threshold of significance of the p-values was set at 0.05. QGis software (version 3.36.3) [31] was used to generate the maps of seroprevalence variation and geolocation of study sites. The administrative region was chosen as the epidemiological unit to represent the variation in seroprevalence.

## 3. Results

Two thousand one hundred and twenty-seven (2127) sera were collected in the whole country in this cross-sectional study. The small ruminant population sampled included 53.12% sheep and 46.87% goats, the majority of which were females (80.44% of the population). According to age, adults represented 81.38% of the entire population and young animals 18.61%. These results provide an overview of the distribution and most relevant factors of these two zoonoses in Senegal. The specificity and sensitivity values for each test, combined with the seropositivity rates obtained, were used to provide true seroprevalence rates for each relevant parameter. However, the sampling strategy was structured by herd and by department, and this may have influenced the seroprevalence observed. Moreover, selected herds were targeted according to their highest probability of exposure to the two viruses. Consequently, the seroprevalence rates obtained are not an estimation of the entire small ruminant population but of a sub-population with a higher risk of exposure to the virus.

### 3.1. Serological Status of Crimean-Congo Hemorrhagic Fever in Small Ruminants in Senegal

The following results showed an overall seroprevalence of 14.1% (IC 95%: 12.5–15.5) for Crimean-Congo hemorrhagic fever in Senegal. High rates of seropositivity were found in adults (16.2%; 95% CI: 14.5–18) and females (15.4%; 95% CI: 13.7–17.1) of small ruminants, in comparison with young animals (4.9%; 95% CI: 2.7–7) and males (8.8%; 95% CI: 6–11.5), respectively. Seroprevalence rates of 16.4% (95% CI: 12.4–18.5) and 11.6% (95% CI: 9.6–13.6) were obtained in sheep and goats, respectively. The Chi-2 test showed significant differences between the variables of age (*p*-value = 8.783 × 10^−9^, sex (*p*-value = 0.0006) and species (*p*-value = 0.001947). Indeed, the groups of adults and sheep were found to be more susceptible to the CCHF virus infection. Logistic regression analyses also showed that the risk of infection was 72% lower in young animals (OR = 0.28; *p*-value < 0.001) than in adults. Similarly, depending on the species, goats were 40% (OR = 1.60; *p*-value < 0.001) less likely to be infected than sheep. Furthermore, the analysis showed that there is no effect of disease in relation to the sex variable (OR = 0.73; *p*-value = 0.11) (Table 1).

**Figure 2:**
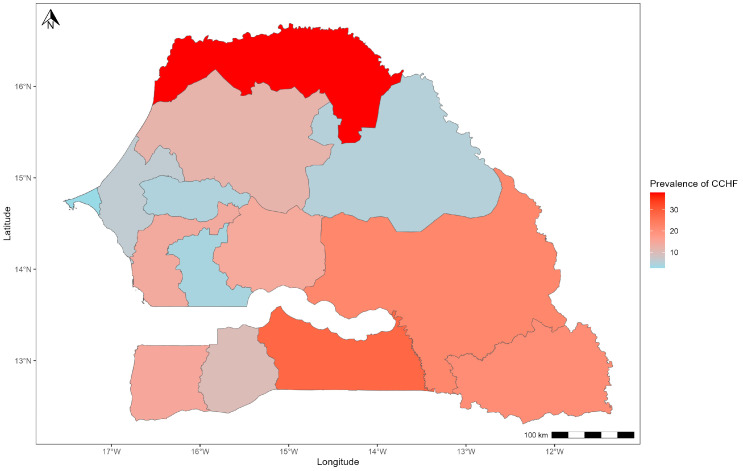
Spatial distribution of CCHFV seroprevalence of small ruminants in Senegal.

According to region, the seropositivity rates obtained showed that the regions of Saint-Louis (38.4%; 95% CI: 30.4–46.2), Kolda (28.3%; 95% CI: 20.9–35.7), Tambacounda (22.2%; 95% CI: 15.8–28.6) and Kédougou (20.9%; 95% CI: 14.4–27.4) were more affected by the CCHFV than the other regions. In addition, the spatial distribution of seroprevalences showed that the virus was distributed differently across areas. In fact, the western (Dakar and Thies) and northeast (Matam) regions were less affected than regions in the south-east (Tambacounda, Kédougou and Kolda) and north-west (Saint-Louis) of Senegal. The same trend observed in the center of the country was found in the southwestern part, in Sédhiou and Ziguinchor, with relatively low seroprevalences (Figure 2).

### 3.2. Serological Status of Rift Valley Fever in Small Ruminants in Senegal

In this study, an overall seroprevalence of 4.4% (95% CI: 3.5–5.3) was found for Rift Valley fever in Senegal. Risk factors such as age, sex and species were assessed, and the results showed higher seropositivity rates in females (5%; 95% CI: 4–6) and adults (5.1%; 95% CI: 4–6.1) of small ruminants compared with males (2%; 95% CI: 0.6–3.3) and young animals (1.6%; 95% CI: 0.3–2.8), respectively. According to species, seroprevalences of 5.4% (95% CI: 4–6.8) and 3.5% (95% CI: 2.4–4.6) were obtained in goats and sheep, respectively. Statistical analyses showed significant differences between the variables of species (*p*-value = 0.007), age (*p*-value = 0.002) and sex (*p*-value = 0.014). The risk factor analysis for RVF showed that the variables of age (OR = 3.70; *p*-value = 0.2) and sex (OR = 1.50; *p*-value = 0.6) had no significant incidence of the occurrence of the disease. On the other hand, according to the species variable, sheep were less exposed to the disease, while the risk of exposure for goats increased by 2.71 (OR = 2.71; *p*-value = 0.040) (Table 2).

**Figure 3:**
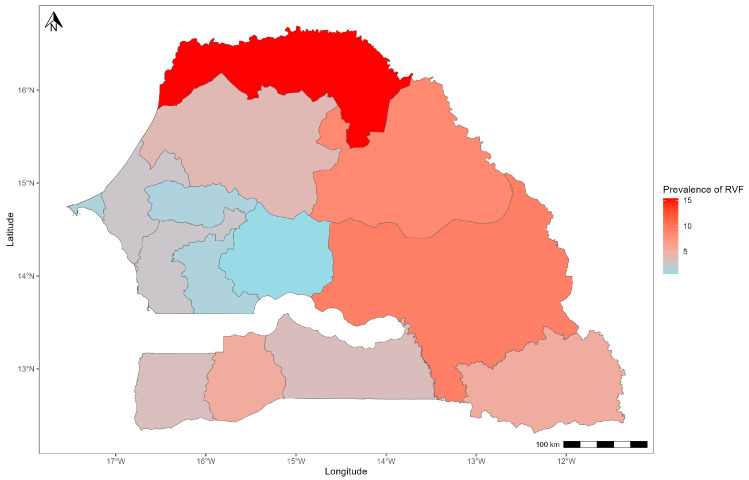
Spatial distribution of RVFV seroprevalence of small ruminants in Senegal.

The spatial distribution of seroprevalences shows that the north and the east of the country are more affected by RVFV. Indeed, higher seropositivity rates were obtained in the regions of Saint-Louis at 15.2% (95% CI: 9.4–20.9), Tambacounda at 9% (95% CI: 4.6–13.4) and Matam at 8.2% (95% CI: 3.9–12.6). The lowest rates of seropositivity were observed in the western and central regions of the country (Figure 3).

## 4. Discussion

There was a real need to study seroprevalence to aid understanding of the distribution and potential risk of CCHF and RFV to livestock across the country in the face of recurrent episodes of epidemics and sporadic cases of these two zoonoses in humans. Several studies have been carried out in Senegal, but these have not considered all regions. Existing research is more focused on the northern part of the country. Our study is the first national cross-sectional serological survey to assess the distribution of the two diseases in the small ruminant populations of Senegal. The sampling strategy adopted in this study enabled surveys in all the regions of Senegal. In each region, several districts were sampled to give a wide spatial distribution and overview of the situation regarding these two diseases in Senegal. Therefore, this research will update the distribution of both viruses and help to improve surveillance strategy. Indeed, maps of prevalence provide information on the serological profile of animals exposed to CCHF and RVF viruses, as well as the most potential areas currently at risk. This information helps to guide surveillance and response to both diseases in specific areas. This study was performed considering the species, sex and age of both viruses. Previous studies have shown that CCHF and RVF prevalences vary as a function of these factors [32,33].

Crimean-Congo hemorrhagic fever is a significant threat to public health and is endemic in Africa, Asia and Europe. Its epidemiology remains poorly documented [34]. In this study, a nationwide cross-sectional survey of small ruminants was carried out, resulting in an estimated overall seroprevalence of 14.1% for the CCHF virus. The results of this study, combined with the human cases of CCHF recently reported in Senegal, attest to an active circulation of the virus. Indeed, Mhamadi et al. [13] reported in their studies in northern Senegal that the CCHF virus is circulating in both human and animal populations. According to age, a higher seroprevalence was found in adults compared to young animals. This result corroborates those [35] in a serological survey carried out in various localities in Senegal. Meanwhile, the results of CCHF virus surveillance in sheep in the north of the country showed that adults’ risk of exposure to the virus increased by 1.26 months compared with young animals [13]. This disparity could be explained by the herd structure in Senegal. Indeed, adults are generally more abundant than younger animals, and given their longevity, are at higher risk of exposure to infective tick bites. According to species, the results showed that CCHF seroprevalence was much higher in sheep than in goats. Our results corroborate those of Mangombi et al. [12], who reported an increased risk of infection in sheep compared with goats in northern Senegal. Similar observations were obtained from studies carried out in Iraq, Saudi Arabia and the Gambia [17,36,37]. In Cameroon, Simo Tchetgna et al. [38] showed that sheep were more susceptible than goats. Another seroprevalence study conducted in Turkiye estimated higher seropositivity in goats than sheep, using a serum neutralization assay [39]. These observations could be explained by the host’s preference for ticks in sheep reported by Badji [40] in Senegal. According to the factor of sex, females seem to be more affected by the disease than males, as observed by Dieye [35]. These results could also be explained by the herd structure, where there are generally more females than males due to the sale of males at feasts and for the breeding requirements of a herd. In addition to age, sex and species, longitude in terms of the distance from the movement location (district) appears to have an influence on the seropositivity of small ruminants [41]. Spatial distribution showed that the seroprevalence of CCHF is higher in northern Senegal. Indeed, Mangombi et al. [12] indicated that the infection of CCHF was prevalent in this part of the country. This northern region neighbors Mauritania and Mali, which are the main commercial conveyors of livestock to Senegal and endemic to the CCHFV [41]. The virus’s introduction to Senegal from a neighboring country was confirmed by the findings of Sene et al. [42]. Indeed, this high seroprevalence may be explained by the relative abundance of CCHF virus vectors (*Hy. rufipes* and *Hy. truncatum*) in this area described by Badji et al. [19], as well as their proximity to Mauritania, where the virus circulates recurrently and almost endemically [43]. On the other hand, the infection rate of ticks collected by Badji et al. [19] in the north was higher than those estimated in the center and south of Senegal. Our findings and previous studies in humans show that the northern Senegal region is the main risk area for CCHF [18]. Given this observation, a longitudinal investigation of the seroprevalence of the CCFHV and its dynamic vectors in these areas will allow for an improvement in our understanding of the epidemiology of the diseases.

Rift Valley fever (RVF) is a zoonotic arbovirus whose outbreak is often associated with a significant increase in the number of mosquito vectors following periods of heavy rainfall [44]. The results obtained in this study showed an overall serological prevalence of RVF of 4.4%, which appears to have decreased compared to the national prevalence of RVF in Senegal three years after the 2013 epizootic, which was estimated at 9.94% [45]. However, RVF still persists in Senegal, and outbreaks were reported in 2003 by the national surveillance network. Outbreaks have also occurred in the Senegal River valley, and numerous studies have shown active RVF virus circulation in many areas across the country [46,47,48]. Risk factor analysis showed that goats are more infected than sheep, which is in line with the results of Hama et al. [32]. However, opposite results were found by Mahmoud and Ali [49] in Egypt, and Blomström et al. [50] and Girard [51] in Mozambique and Comoros. This disparity could be explained by differences in vector species in each zone and their trophic or host preferences. Depending on the age of the animals, results have shown that adults exhibit higher seroprevalence of RVF virus infection than young animals. According to Thiongane et al. [52], young animals have a significantly lower antibody prevalence than adults, corroborating our results. Mhamadi et al. [47] described the presence of significantly higher RVF IgG antibodies in adult sheep, and all the animals that seroconverted to RVFV during their study were adults. The increase in temperature and vegetation density are important environmental risk factors and should, therefore, be taken into account in subsequent quantitative analyses of RVF. The spatial distribution of seroprevalences obtained for RVF shows that the northern axis towards the southeast of the country is more affected by the disease than the west and center of the country. Saint-Louis and Tambacounda, with 15.2% (95% CI: 9.4–20.9) and 9% (95% CI: 4.6–13.4), respectively, had the highest prevalence. Our findings corroborate those of Sankhe et al. [18] in humans. The findings show that the risk of exposure is high for the northern and eastern human populations. Entomological risk factors, including temperature and rainfall, may explain the high seroprevalence in the eastern part of the country. In fact, previous studies have described the diversity and abundance of mosquito vectors in the north and south of Senegal [53,54], which may be influenced by high rainfall levels and the availability of water and irrigation at regular intervals. Indeed, Biteye et al. [53] showed that *Culex tritaeniorhynchus* and *Mansonia uniformis* were the most abundant and frequent species in northern Senegal (River Delta and Senegal River Valley). These observations are similar to those of Fall et al. [55] and Fall et al. [56] at Ross Béthio (in the north).

Additional investigation into the vector’s dynamics and the seroprevalence of RVF in this region will be provided as supplementary data to better understand the epidemiology of this virus in this part of Senegal. The lack of information on the investigation of the CCHFV in small ruminant populations can be considered a limitation of this study. Indeed, with the expected prevalence data, the sample size estimation could be more accurate. The Elisa methods used in this study are valid techniques, and the commercial kits are highly specific and sensitive tests with values close to 100%. While the VNT method is recognized as the gold standard test, the large sample size and the very high level of biosecurity required for Rift and Crimea-Congo viruses made the VNT method difficult to apply.

## 5. Conclusions

For the first time, a nationwide study has provided a baseline for the Crimean-Congo hemorrhagic fever and Rift Valley fever in small ruminants in Senegal and has revealed several significant aspects. The results of this work on the potential hosts of the CCHF virus associated with recent recorded human cases demonstrate the active circulation of the virus in Senegal. This work carried out here in the context of RVF, confirms the endemicity of the virus and its distribution on a national scale. The risk factors identified in this study indicate that the age, species and sex of animals are key factors in determining exposure to the two viruses. In general, these two zoonoses present broadly similar serological profiles in Senegal. In fact, the north of the country is more at risk from both RVFV and CCHFV. The southeastern part of Senegal presents a particular challenge, with high rates of seropositivity that are not associated with human cases of Crimean-Congo or Rift Valley fever. Additional studies, including the isolation and characterization of the viruses from ticks, mosquito vectors and infected animals and longitudinal studies in sheep and goats, are necessary to further understand the epidemiology of both viruses.

## Figures and Tables

**Table 1:** Seroprevalences and logistic regression analysis of CCHFV risk factors in small ruminants in Senegal.

Risk Factors		Total Samples	Total Positive	Apparent Prevalence (%)	True Prevalence (%)	*p*-Value Chi2 Test	OR (*p*-Value)
Age	Adult	1731	278	16.06	16.2	8.783 × 10^−9^	0.28 (<0.001)
	Young	396	19	4.79	4.9		
Sex	Female	1711	261	15.25	15.4	0.0006624	0.73 (=0.11)
	Male	416	36	8.65	8.8		
Species	Sheep	1130	183	16.19	16.4	0.001947	1.60 (<0.001)
	Goats	997	114	11.43	11.6		

**Table 2:** Seroprevalences and logistic regression analysis of RVFV risk factors in small ruminants in Senegal.

Risk Factors		Total Samples	Total Positive	Apparent Prevalence (%)	True Prevalence (%)	*p*-Value Chi2 Test	OR (*p*-Value)
Age	Adult	1731	91	5.25	5.1	0.00269	3.70 (=0.2)
	Young	396	7	1.76	1.6		
Sex	Female	1711	89	5.20	5.0	0.01409	1.50 (=0.6)
	Male	416	9	2.16	2.0		
Species	Sheep	997	56	5.61	5.4	0.007836	2.71 (=0.04)
	Goats	1130	42	3.71	3.5		

## Data Availability

For reasons of confidentiality, the data used in this study are not published. Selected information may be shared with interested upon request and approval of the institute and herd owners.
